# Supplementary material for: The architecture of transmembrane and cytoplasmic juxtamembrane regions of Toll-like receptors
Source: Nat Commun. 2023 Mar 17;14:1503. doi: 10.1038/s41467-023-37042-6 (PMC10023784; doi:10.1038/s41467-023-37042-6)
Supplement: Supplementary file 1 — Supplementary Information [file 41467_2023_37042_MOESM1_ESM.pdf]

The Architecture of Transmembrane and Cytoplasmic Juxtamembrane regions of Toll-like receptors.

Kornilov F.D.[1,2,#], Shabalkina A.V.[1,2,#], Cong Lin[3,#], Volynsky P.E.[1,4], Kot E.F.[1,2], Kayushin A.L.[1], Lushpa V.A.[1,2], Goncharuk M.V.,[1] Arseniev A.S.[1], Goncharuk S.A.[1,2,&], Xiaohui Wang[3,5,&], Mineev K.S.[1,2,&]

[1]Shemyakin-Ovchinnikov Institute of Bioorganic Chemistry, Moscow, 117997, Russia

[2]Moscow Institute of Physics and Technology, Dolgoprudny, 141701, Russia

[3]Laboratory of Chemical Biology, Changchun Institute of Applied Chemistry, Chinese Academy of Sciences, Changchun, Jilin, 130022, China

[4]Institute of Cytology of Russian Academy of Sciences, Tikhoretsky 4, 194064 Saint Petersburg, Russia.

[5]School of Applied Chemistry and Engineering, University of Science and Technology of China, Hefei, Anhui, 230026, China

[#]These authors contributed equally to the work

[&]These authors jointly supervised the work, e-mail: ms.goncharuk@gmail.com, xiaohui.wang@ciac.ac.cn, mineev@nmr.ru

Supplementary Information

Supplementary Table 1. Conditions used to determine the spatial structures of the proteins

Subject	**TLR2tmjm**	**TLR3tmjm**	**TLR5tmjm**	**TLR9tmjm**
Membrane mimetic	bicelles DMPC/DMPG/DHPC (DMPC:DMPG=4:1), q*=0.4	micelles DPC	micelles DPC	micelles LPPC/LPPG (PC:PG=3:1)
LPR	500	500	220	160
Concentration	200μM	270μM	1mM	1mM
Temperature	45°C	45°C	40°C	45°C
pH	7.0	7.0	6.0	7.0
Buffer	20mM KPi, 4 mM TCEP, 0.05% NaN_3, 1 mM TSP			

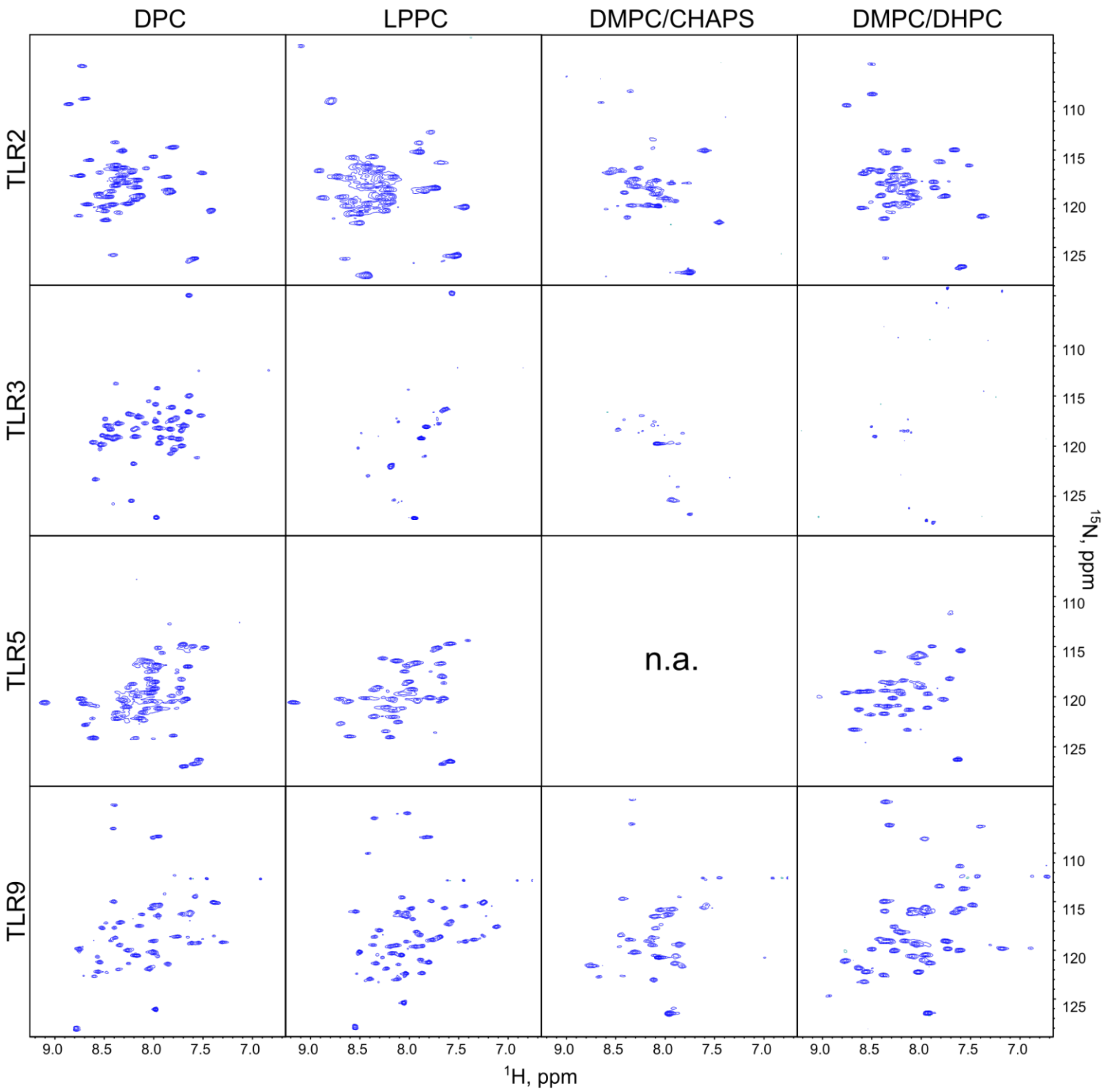

Supplementary Figure 1. ^{1}H^{15}N-HSQC of TLR2tmjm, TLR3tmjm, TLR5tmjm and TLR9tmjm in different membrane mimetics. For the particular peptide in different mimetics spectra were recorded with the same temperature and pH, the approximately equal concentration of the peptide, equal parameters of the NMR experiment, and with the maximum lipid-to-protein ratio that is appropriate for a particular mimetic. The spectra are shown with the same widths in ^{1}H and ^{15}N directions and with the same contour parameters.

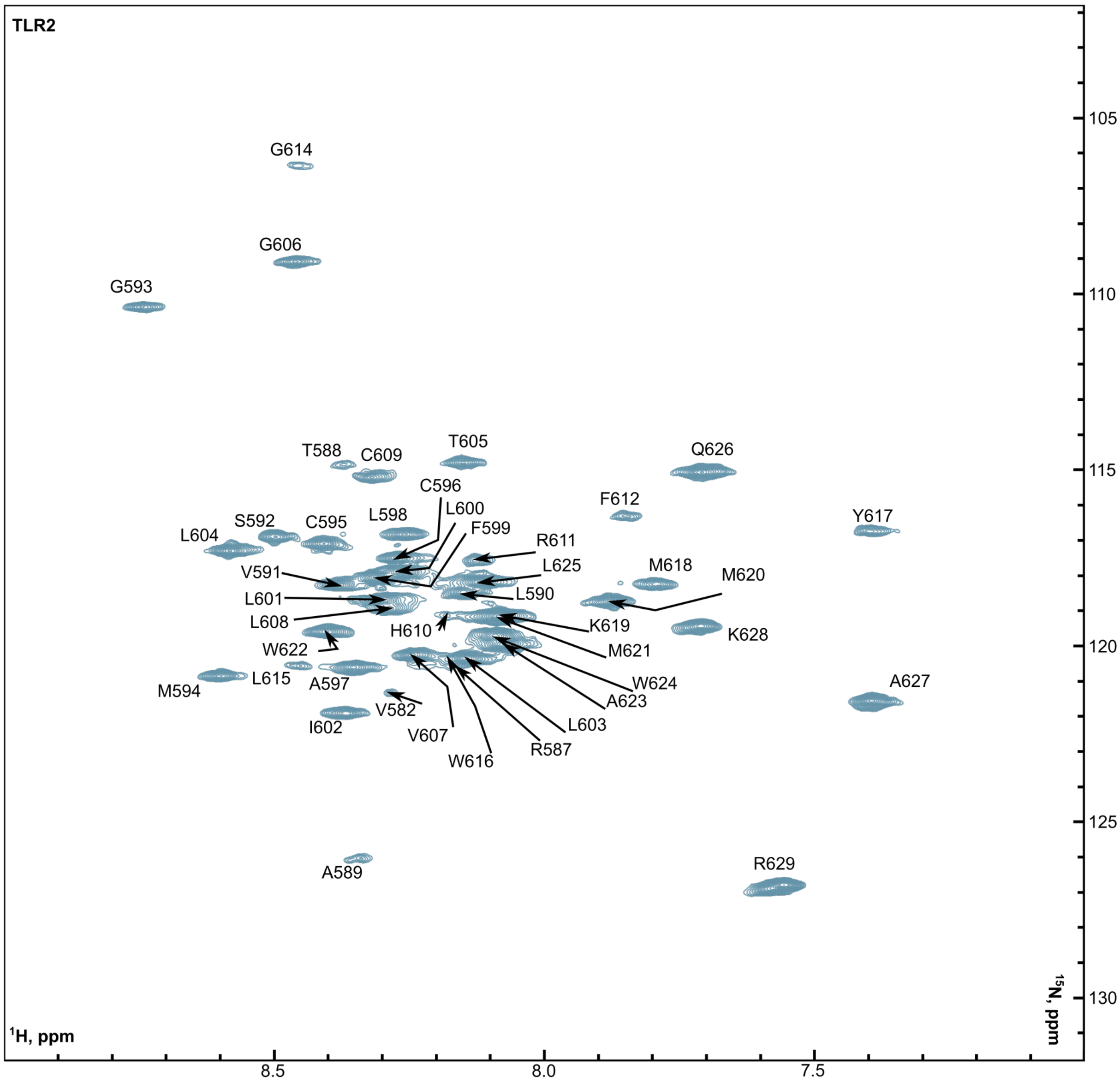

Supplementary Figure 2. $^{1}H^{15}N$-TROSY-HSQC of TLR2tmjm in DMPC/DMPG/DHPC bicelles (DMPC:DMPG=4:1), q=0.4, T=318K, pH=7.0

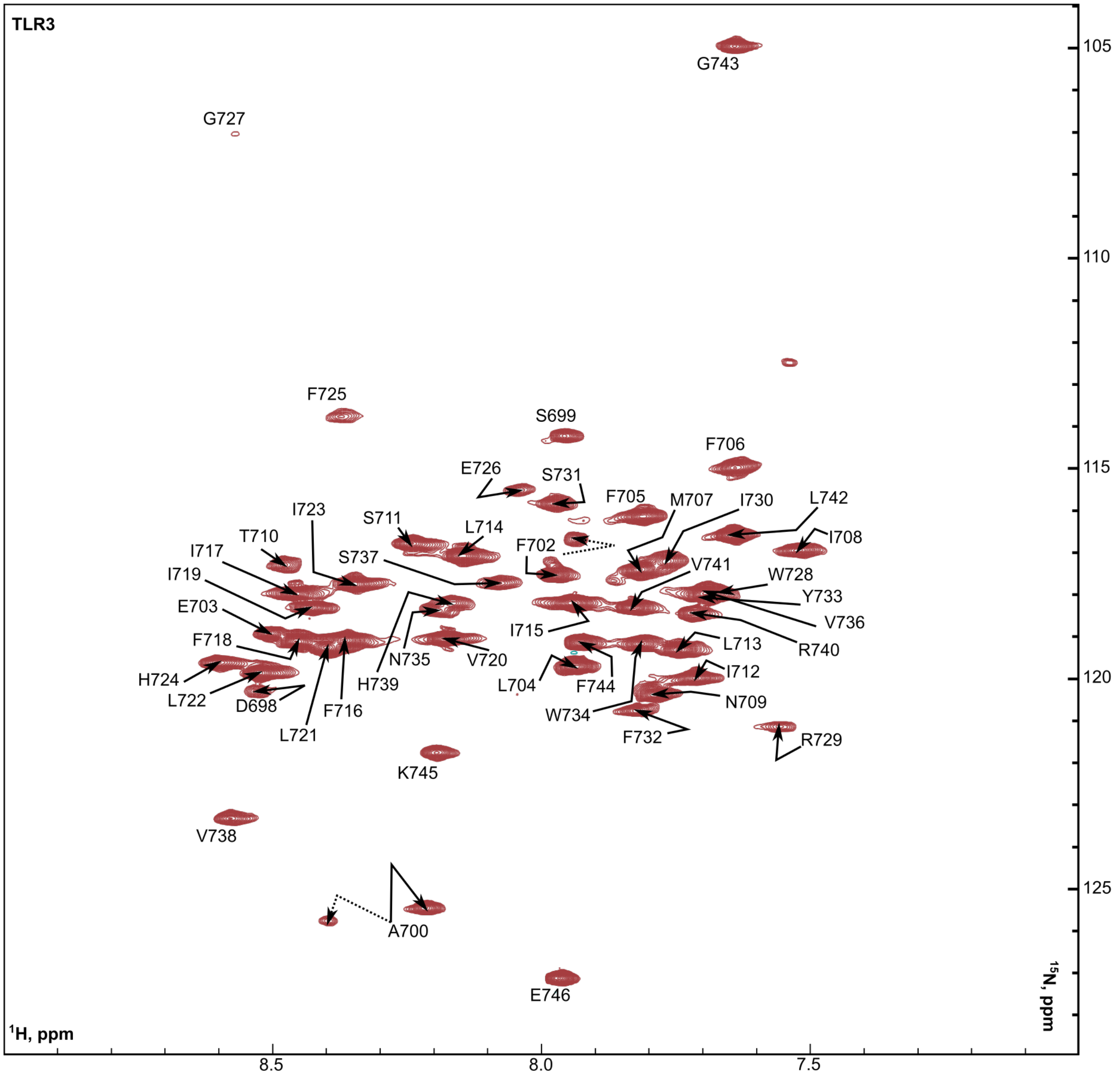

Supplementary Figure 3. $^{1}H^{15}N$-TROSY-HSQC of TLR3tmjm in DPC micelles, T=318K, pH=7.0

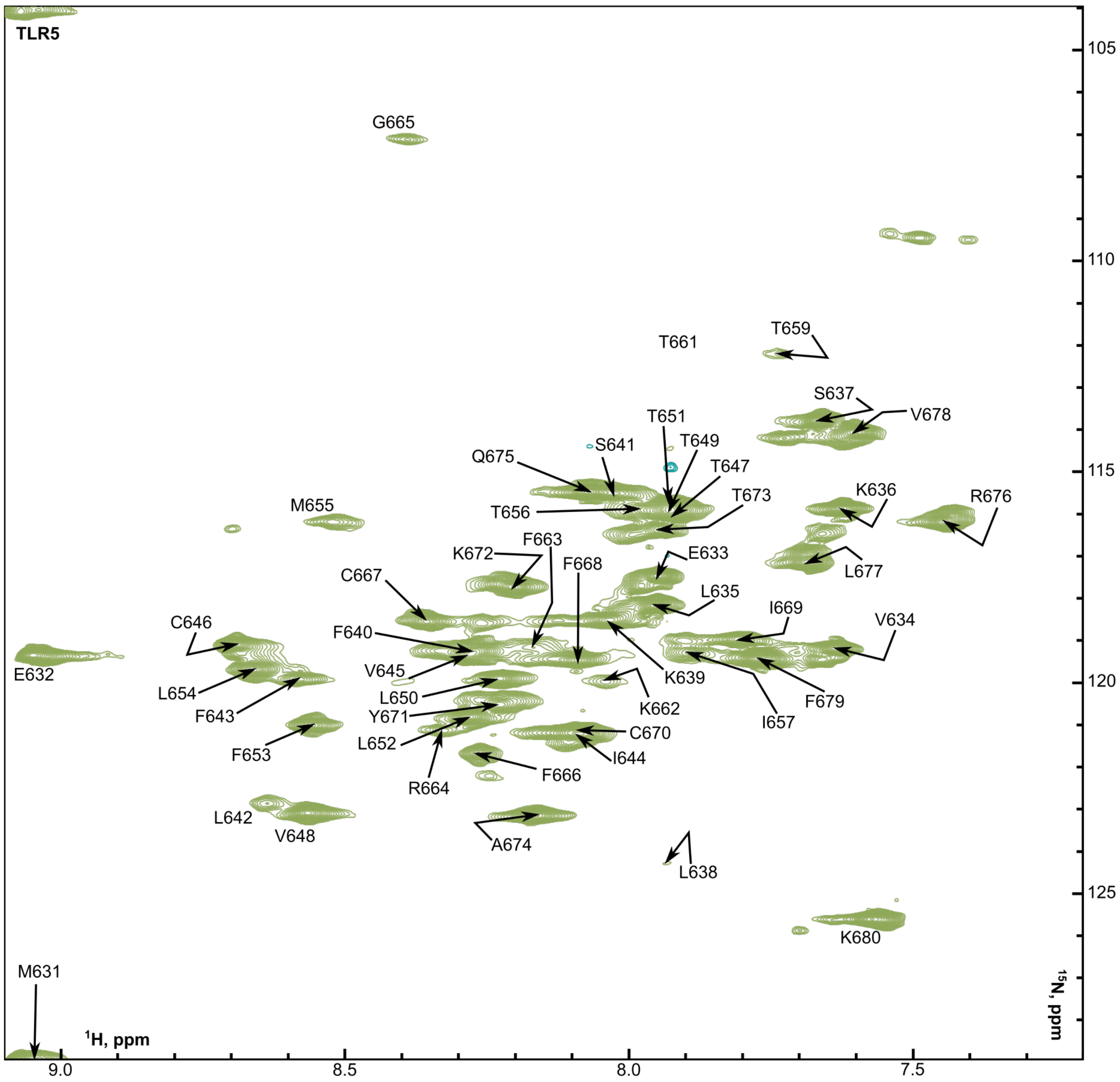

Supplementary Figure 4. $^{1}H^{15}N$-TROSY-HSQC of TLR5tmjm in DPC micelles, T=313K, pH=6.0

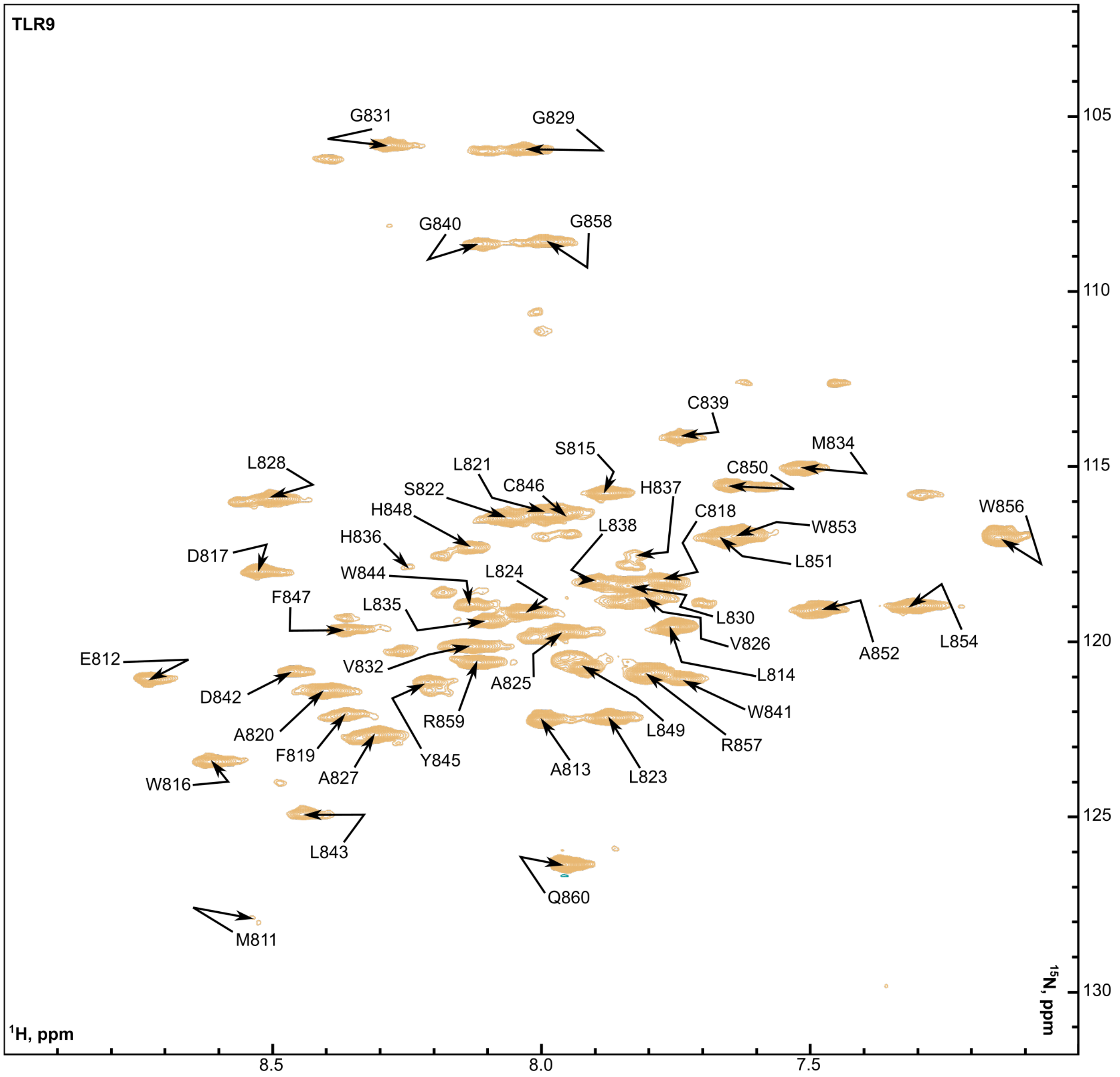

Supplementary Figure 5. ^{1}H^{15}N-TROSY-HSQC of TLR9tmjm in LPPC/LPPG micelles (LPPC:LPPG=3:1), T=318K, pH=7.0

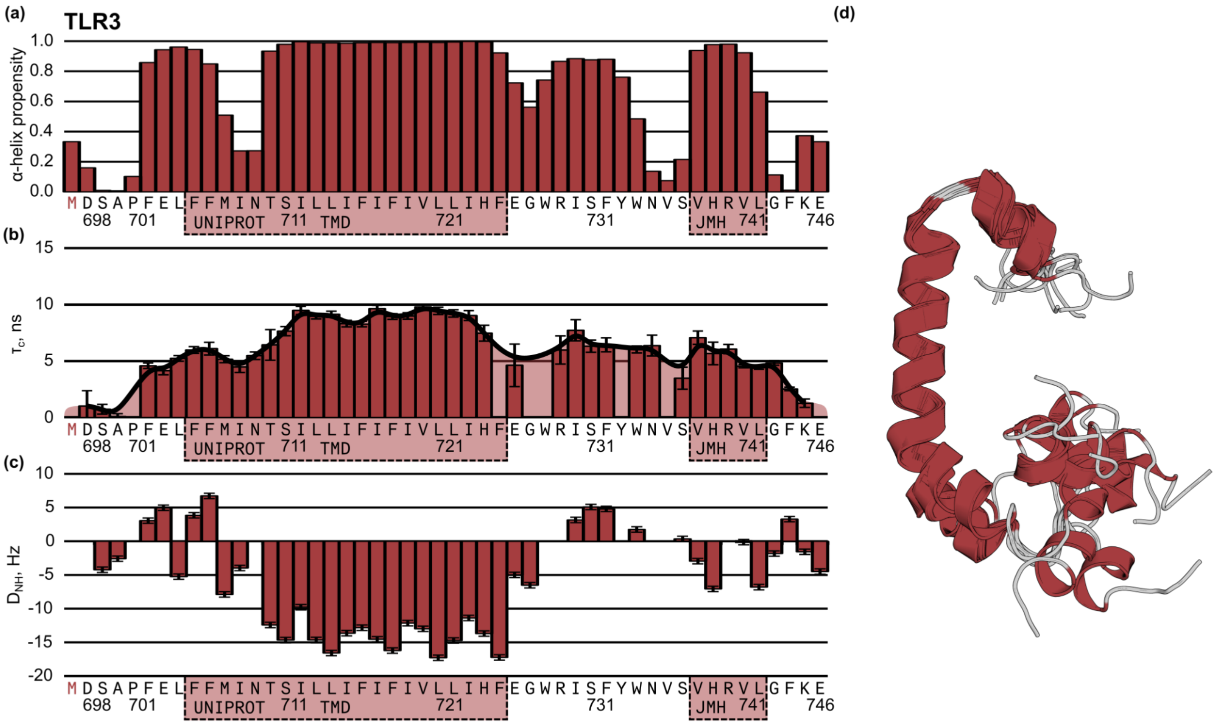

Supplementary Figure 6. Spatial structure of TLR3tmjm in DPC micelles. **A** - the probability of α-helix conformation of the amino acid residues. **B** - values of τ_C, correlation time of rotational diffusion, for the individual amide groups of the amino acid residues. The error-bars were estimated based on the noise level of the spectra. **C** - magnitudes of residual dipolar couplings ($^1D_{NH}$). The sequence is numbered according to UNIPROT. The red boxes denote the parts of the TLR3tmjm: the region that is annotated in UNIPROT as the transmembrane domain and the region that forms the juxtamembrane α-helix (JMH). The red color of the first Met indicates that it is not included in the original sequence of TLR3. The error-bars correspond to the spectral resolution. **D.** The ten best NMR structures that are superimposed on the backbone atoms of transmembrane α-helix. Source data are provided as a Source Data file.

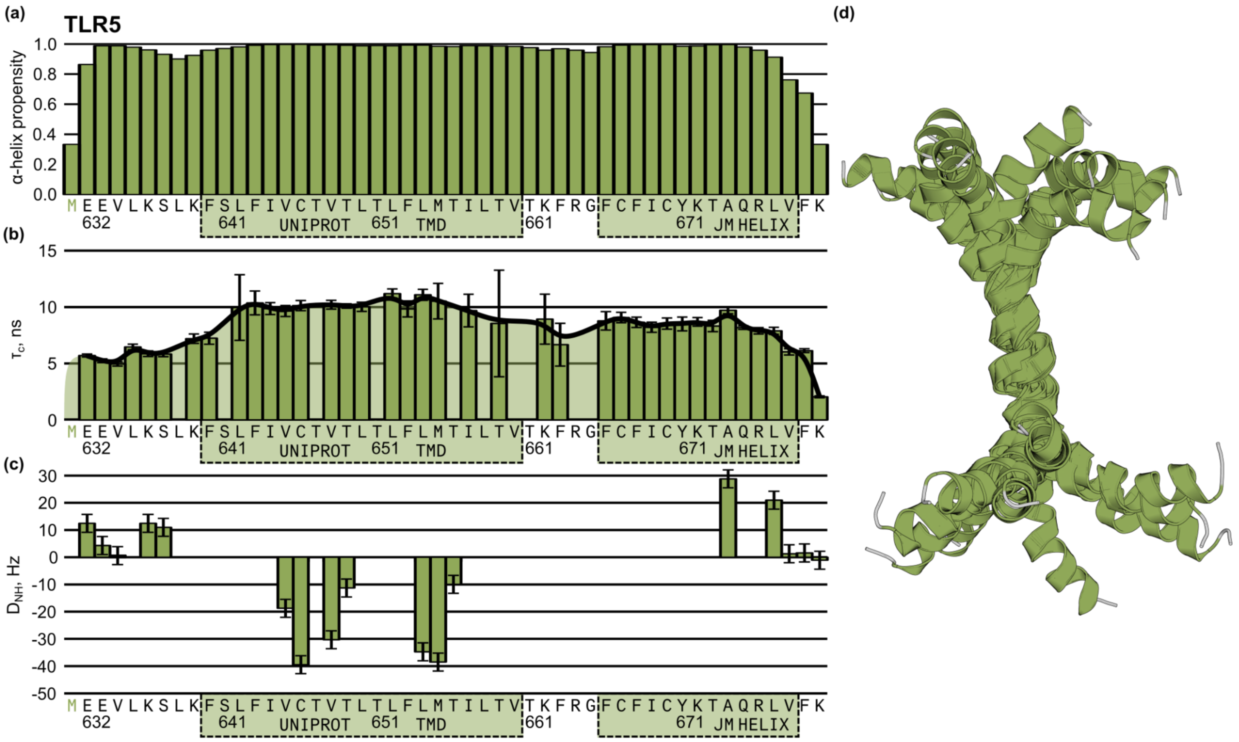

Supplementary Figure 7. Spatial structure of TLR5tmjm in DPC micelles. **A** - the probability of α-helix conformation of the amino acid residues. **B** - values of τ_C, correlation time of rotational diffusion, for the individual amide groups of the amino acid residues. The error-bars were estimated based on the noise level of the spectra. **C** - magnitudes of residual dipolar couplings ($^1D_{NH}$). The sequence is numbered according to UNIPROT. The green box denotes the region that is annotated in UNIPROT as the transmembrane domain. The green color of the first Met indicates that it is not included in the original sequence of TLR5. The error-bars correspond to the spectral resolution. **D.** The ten best NMR structures that are superimposed on the backbone atoms of transmembrane α-helix. Source data are provided as a Source Data file.

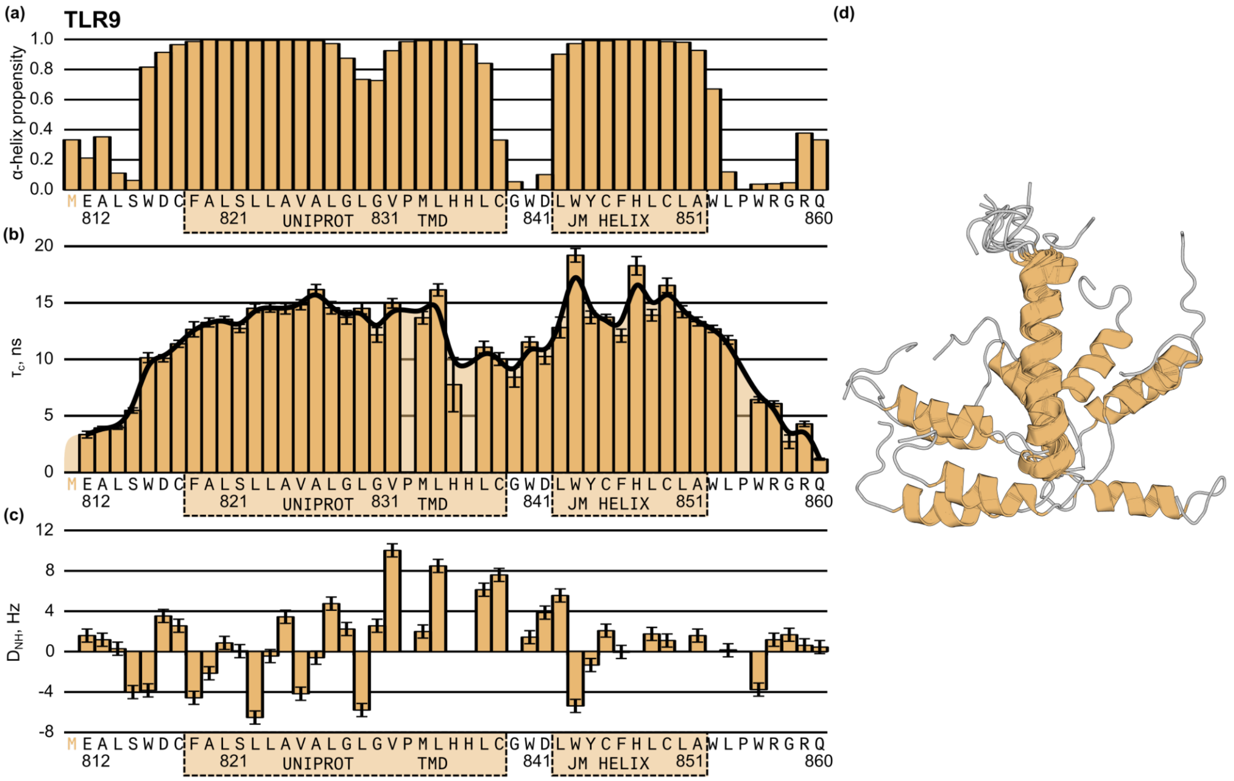

Supplementary Figure 8. Spatial structure of TLR9tmjm in LPPC/LPPG micelles (LPPC:LPPG=3:1). The **A** - the probability of α-helix conformation of the amino acid residues. **B** - values of τ_C, correlation time of rotational diffusion, for the individual amide groups of the amino acid residues. The error-bars were estimated based on the noise level of the spectra. **C** - magnitudes of residual dipolar couplings ($^1D_{NH}$). The sequence is numbered according to UNIPROT. The yellow boxes denote the parts of theTLR9tmjm: the region that is annotated in UNIPROT as the transmembrane domain and the region that forms juxtamembrane α-helix. The yellow color of the first Met indicates that it is not included in the original sequence of TLR3. The error-bars correspond to the spectral resolution. **D.** The ten best NMR structures that are superimposed on the backbone atoms of transmembrane α-helix. Source data are provided as a Source Data file.

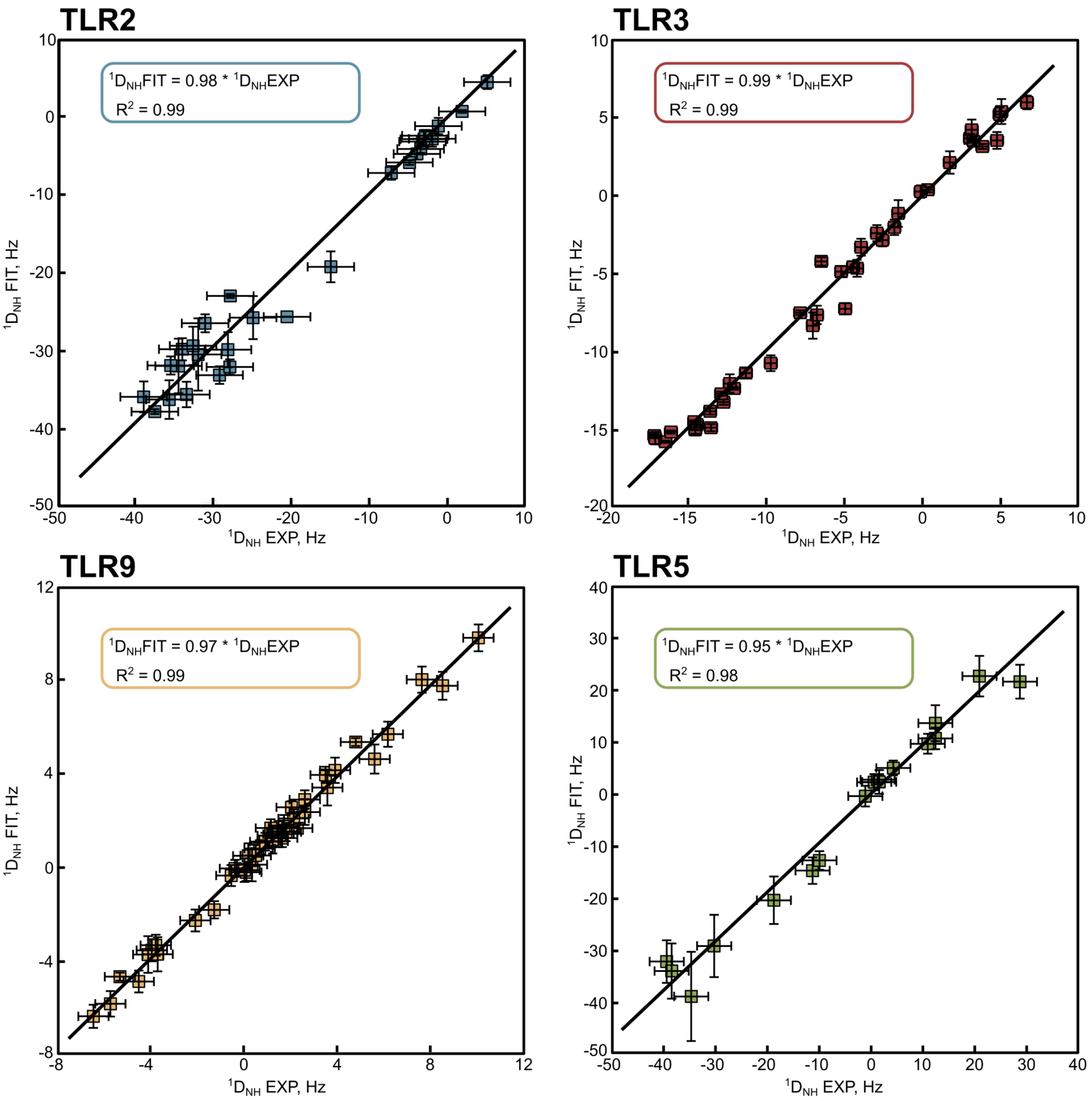

Supplementary Figure 9. Singular value decomposition fit of the $^{1}D_{NH}$ RDCs to the refined NMR structures for TLR2tmjm, TLR3tmjm, TLR5tmjm, TLR9tmjm. Data are presented as mean values +/- standard deviation corresponding to fitting of NMR structures (y-axis) and spectral resolution of the spectra (x-axis). Source data are provided as a Source Data file.

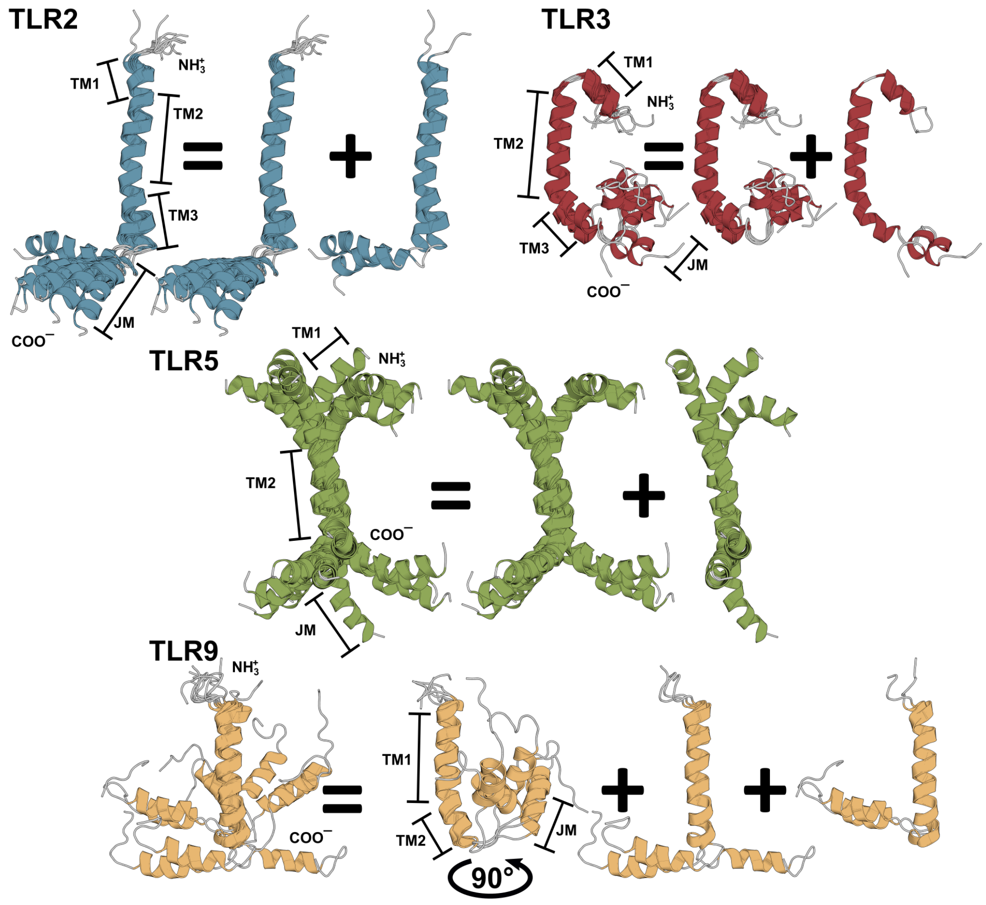

TLR2	TM2	TM3	JM
TM1	168.4°±1.9°	169°±5°	106°±16°
TM2		169°±4°	100°±10°
TM3			101°±17°

TLR3	TM2	TM3	JM
TM1	50°±4°	18°±8°	100°±30°
TM2		130°±6°	65°±30°
TM3			80°±30°

TLR5	TM2	JM
TM1	100°±20°	60°±40°
TM2		100°±30°

TLR9	TM2	JM
TM1	150°±7°	60°±20°
TM2		70°±20°

Supplementary Figure 10. Ten best NMR structures of TLR2tmjm in DMPC/DMPG/DHPC bicelles, TLR3tmjm in DPC micelles, TLR5tmjm in DPC micelles and TLR9tmjm in LPPC/LPPG micelles. Each set of structures was superimposed over the backbone atoms of the transmembrane α-helix and was clustered by relative positions of α-helices. The average values of angles (with standard deviations) between the α-helices are provided in the tables. To calculate

the angles each α-helix was approximated by a cylinder and angles between the axes of the cylinders were found.

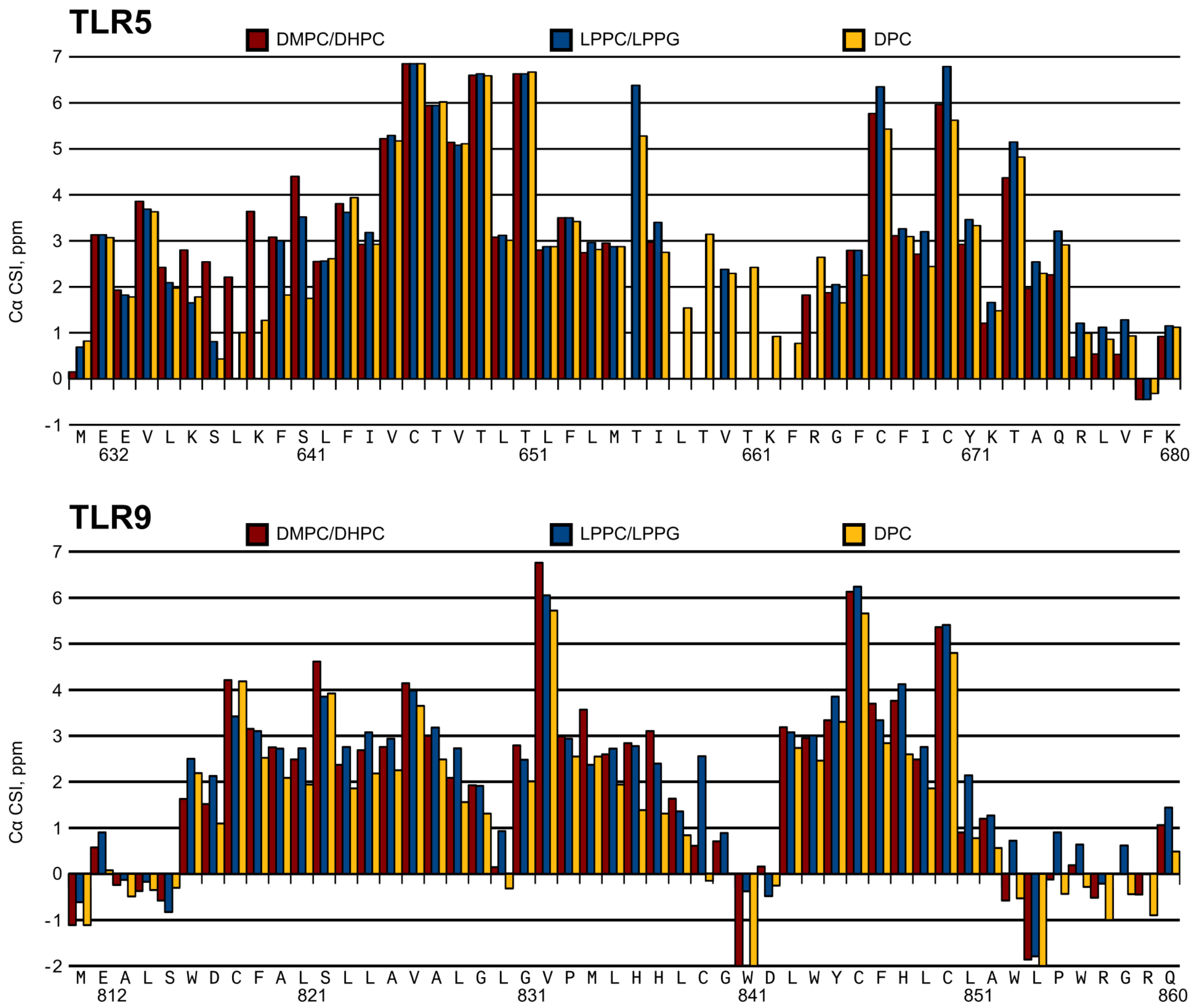

Supplementary Figure 11. Secondary chemical shifts of Cα atoms of TLR5tmjm and TLR9tmjm in various membrane mimetics: DMPC/DHPC (red), LPPC/LPPG (blue) and DPC (yellow). Source data are provided as a Source Data file.

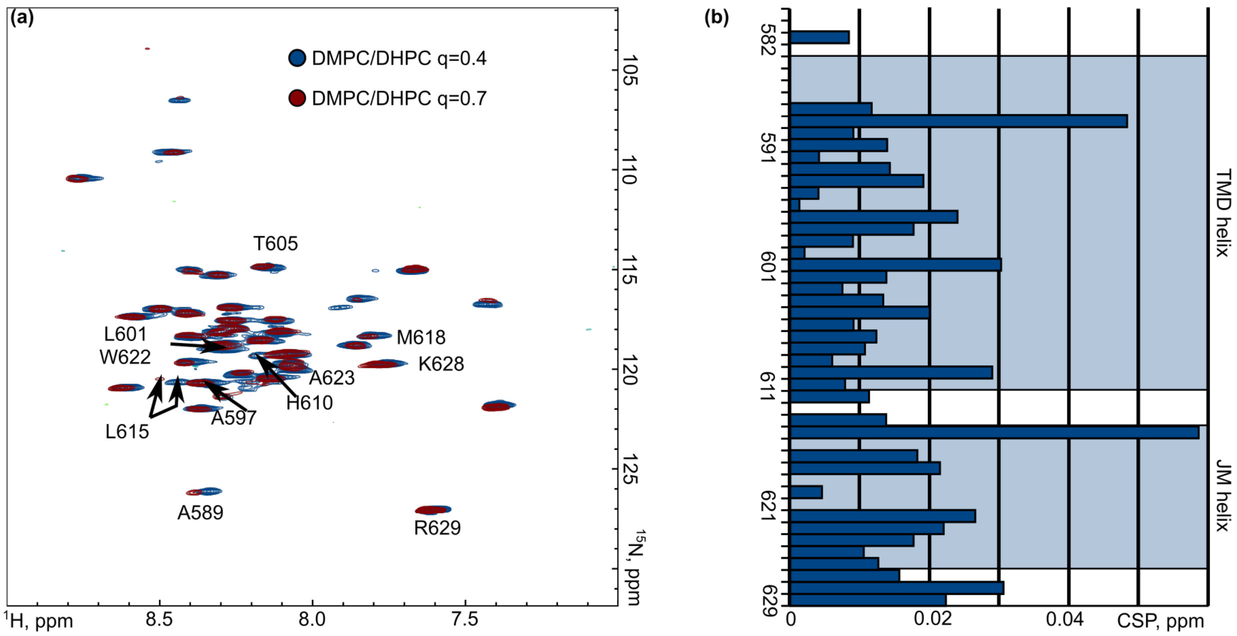

Supplementary Figure 12. Comparison of TLR2tmjm in DMPC/DHPC bicelles with q=0.4 and q=0.7. **A** - The superimposition of $^{1}H^{15}N$-HSQC spectra is shown. The spectrum at q=0.4 is painted blue and at q=0.7 is painted red. **B** - The generalized chemical shift perturbations (CSP) are shown for the individual residues of the protein. CSPs were calculated as follows: $\sqrt{\Delta CS(H\ ppm)^2 + \Delta CS(N\ ppm)^2/10^2}$. Residues with CSPs exceeding 0.02 ppm are indicated on the spectrum. Source data are provided as a Source Data file.

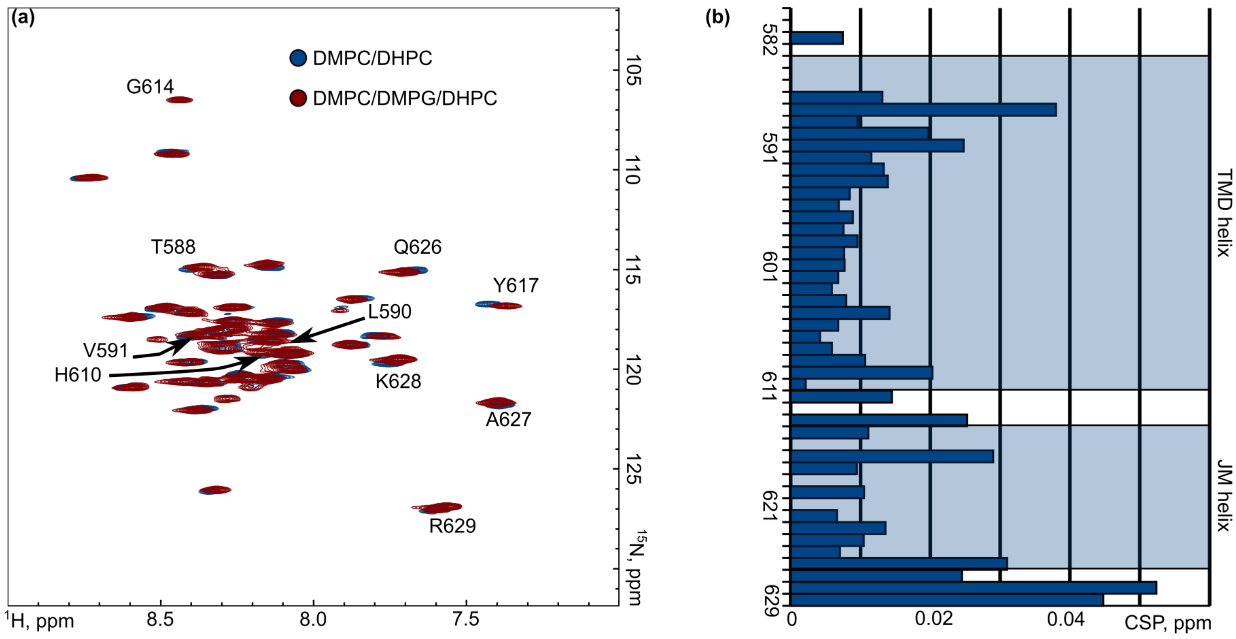

Supplementary Figure 13. Comparison of TLR2tmjm in DMPC/DHPC and DMPC/DMPG/DHPC (DMPC:DMPG=4:1) bicelles. **A** - The superimposition of $^1H^{15}N$-HSQC spectra is shown. $^1H^{15}N$-HSQC of TLR2tmjm in DMPC/DHPC bicelles is painted blue and in DMPC/DMPG/DHPC bicelles is painted red. **B** - The generalized chemical shift perturbations (CSP) are shown for the individual residues of the protein. CSPs were calculated as follows: $\sqrt{\Delta CS(H\,ppm)^2 + \Delta CS(N\,ppm)^2/10^2}$. Residues with CSPs exceeding 0.02 ppm are indicated on the spectrum. Source data are provided as a Source Data file.

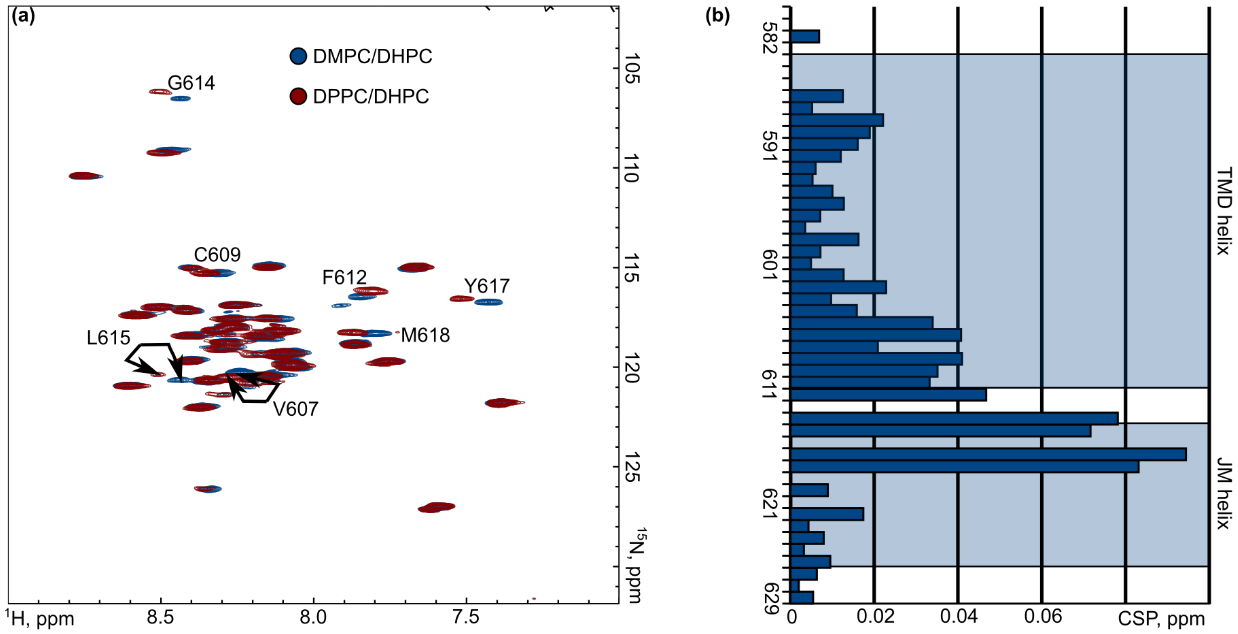

Supplementary Figure 14. Comparison of TLR2tmjm in DMPC/DHPC and DPPC/DHPC bicelles. **A** - The superimposition of $^{1}H^{15}N$-HSQC spectra is shown. $^{1}H^{15}N$-HSQC of TLR2tmjm in DMPC/DHPC bicelles is painted blue and in DPPC/DHPC bicelles is painted red. **B** - The generalized chemical shift perturbations (CSP) are shown for the individual residues of the protein. CSPs were calculated as follows: $\sqrt{\Delta CS(H\ ppm)^2 + \Delta CS(N\ ppm)^2/10^2}$. Residues with CSPs exceeding 0.04 ppm are indicated on the spectrum. Source data are provided as a Source Data file.

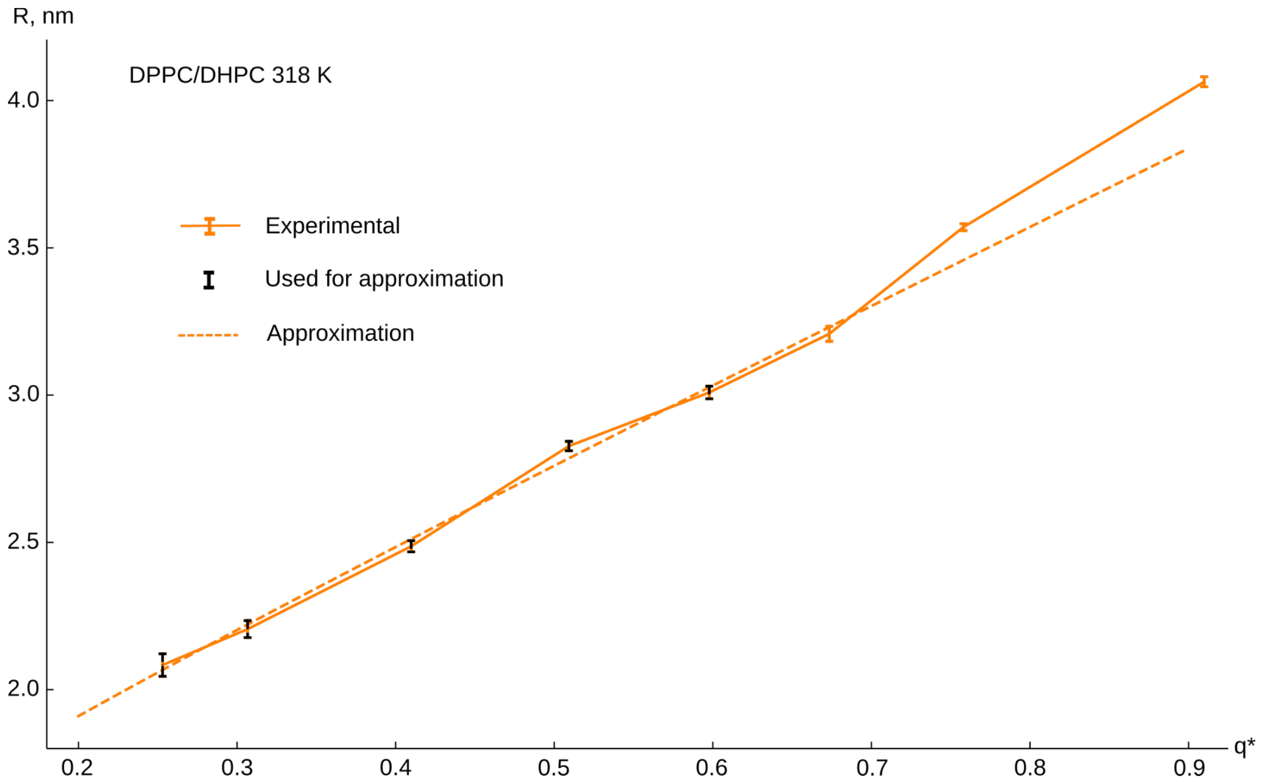

Supplementary Figure 15. DPPC/DHPC bicelles radii at 318K plotted as a function of the effective lipid-detergent ratio (q*) calculated as $[DPPC]/([DHPC]_{total} - [DHPC]_{free})$. The dashed line shows the approximation of the first five experimental points (black) with the ideal bicelle model for a semitoroidal rim[1]. This model determines the radius of a bicelle R as a function of q*:

$$R = r_{\perp} + \frac{r_{\perp} q*}{4\lambda}\left[\pi + \left(\pi^2 + \frac{32\lambda}{3q*}\right)^{1/2}\right].$$

Here $r_{\perp}$ is the thickness of the bicelle rim formed by detergent and λ is the volume ratio of DHPC molecule over DPPC molecule. Error bars represent the standard deviation (SD) calculated using the Monte-Carlo method. Namely, raw diffusion coefficients were obtained from the approximation as D±SD(D), then a set of 500 normally distributed values with the mean value of D and standard deviation of SD(D) were generated and 500 corresponding R values were calculated. Mean values and SDs of each of these sets are plotted.

Experimental data were approximated with $\lambda=0.66\pm0.06$ and $r_{\perp}=1.09\pm0.05$ nm which is very close to the value obtained earlier for DMPC/DHPC bicelles[2]. This indicates that the DPPC/DHPC mixture forms bicelle-like particles that follow the ideal bicelle model. Source data are provided as a Source Data file.

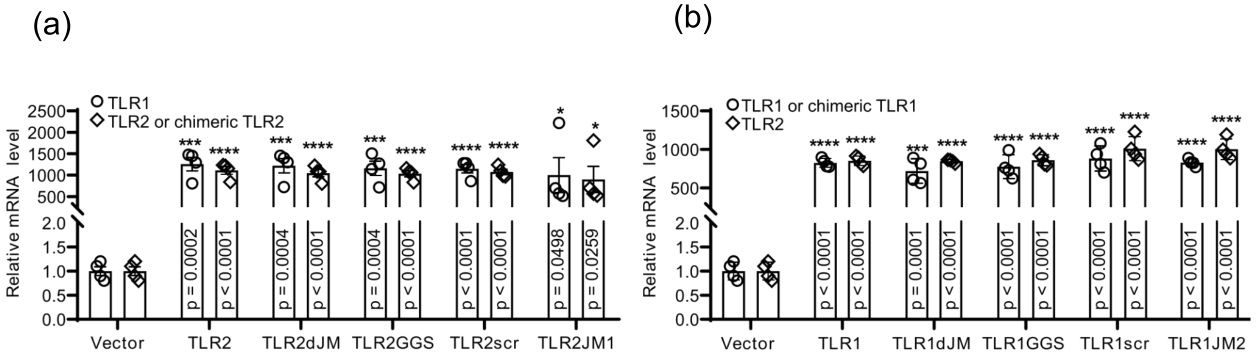

Supplementary Figure 16. A - TLR1 and TLR2 mRNA levels are assessed by qRT-PCR in HEK Blue 293 cells co-expressing human TLR1 and the wild-type or chimera of TLR2. **B** - TLR1 and TLR2 mRNA levels are assessed by qRT-PCR in HEK Blue 293 cells co-expressing human TLR2 and the wild-type or chimera of TLR1. Data are represented as mean ± SD (n = 4). Statistical significance according to the unpaired two-tailed Student's t-test is indicated as follows: * - p < 0.05, *** - p < 0.001, **** - p < 0.0001 with respect to the negative control. Source data are provided as a Source Data file.

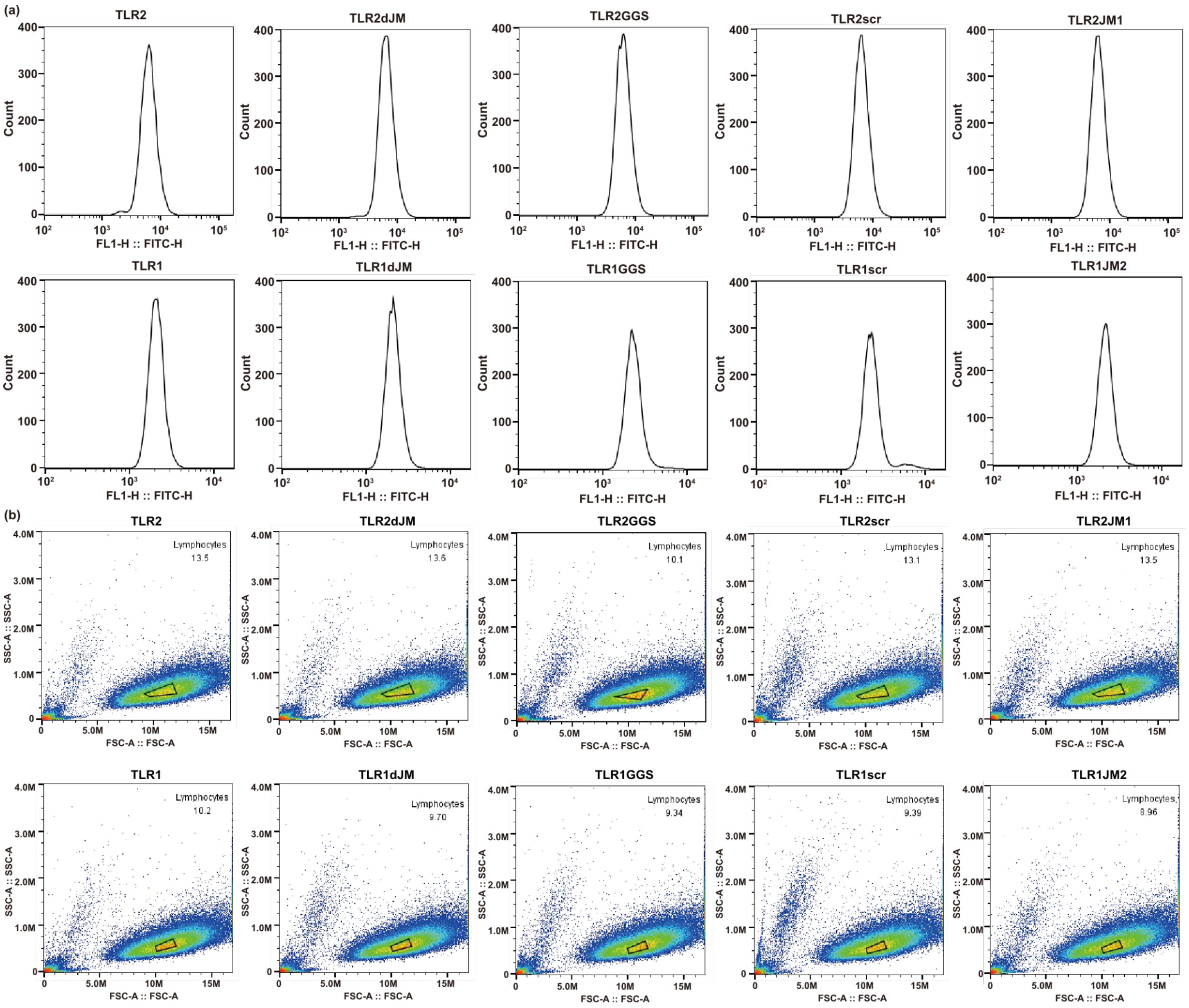

Supplementary Figure 17. A - The membrane localization is monitored by flow cytometry in HEK Blue 293 cells expressing the wild-type or chimera of human TLR1 and TLR2 (titles of chimeric constructs correspond to the titles in Figure 6 in the main text). **B** - Flow cytometry gating strategy for TLR1 or TLR2 staining on viable cells by FSC-A/SSC-A scatter plots. Source data are provided as a Source Data file.

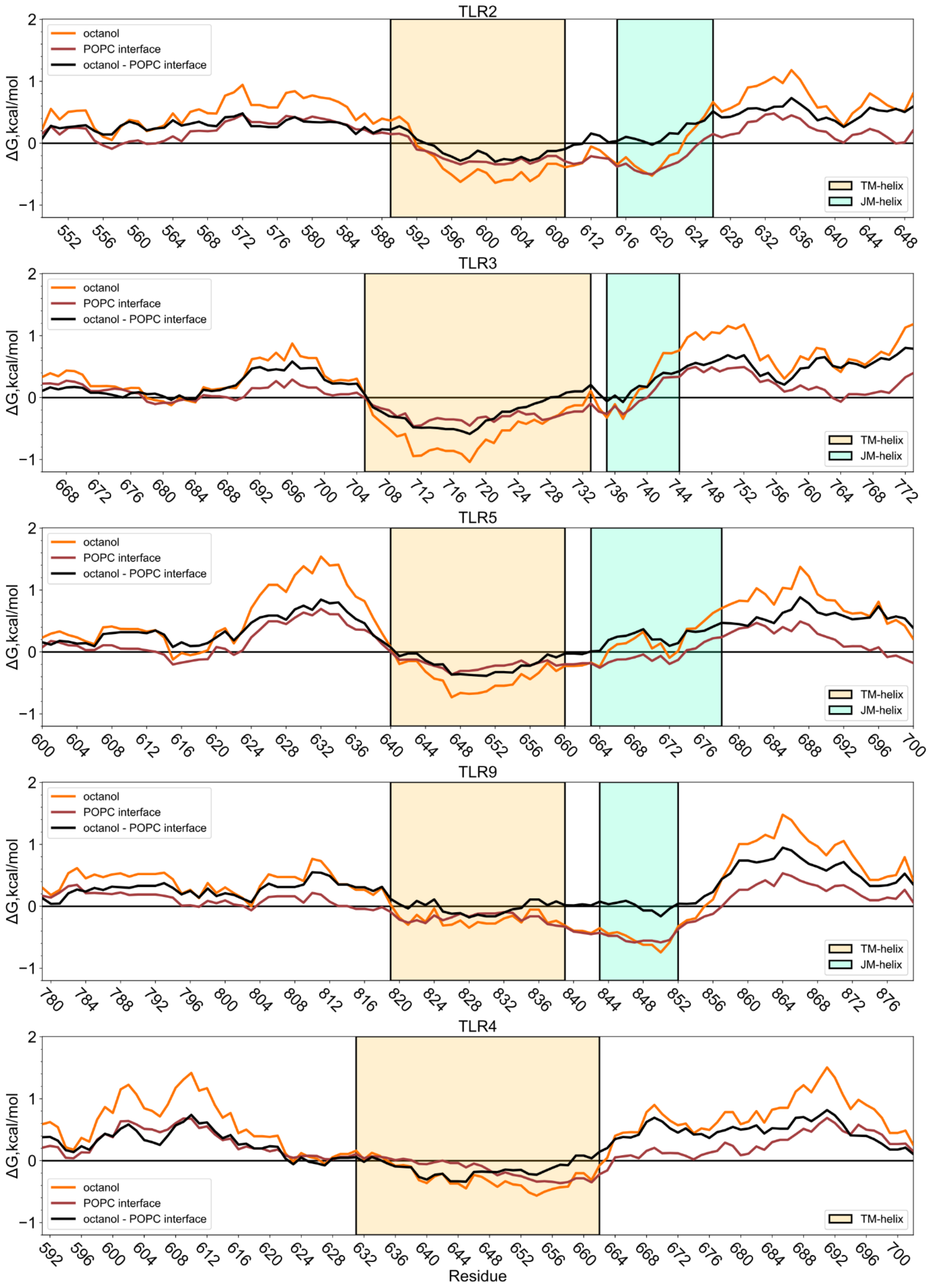

Supplementary Figure 18. Whole-residue hydropathy plots for TLRs, for which the spatial structures of transmembrane and cytoplasmic juxtamembrane regions were determined. For each amino acid, we calculated the average hydrophobicity (the frame equals 15 amino acids) according to White-Wimley scale[3] that shows the free energies of transferring amino acids from water to POPC interface (POPC interface), from water to n-octanol (octanol) and difference between these two scales (octanol - POPC interface). Yellow and cyan backgrounds indicate transmembrane (TM-helix) and juxtamembrane (JM-helix) helices according to this work and work on TLR4[4]. Source data are provided as a Source Data file.

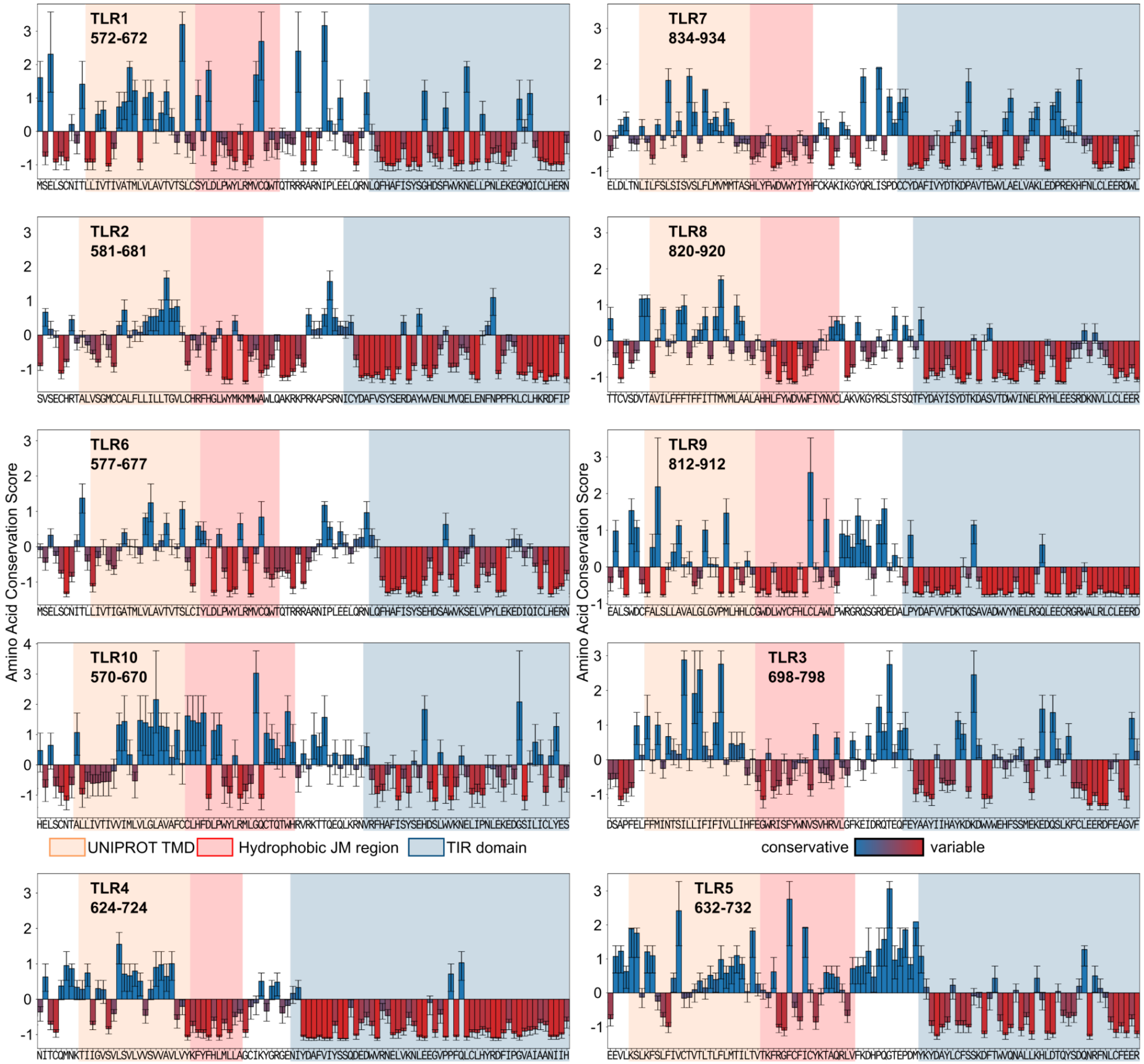

Supplementary Figure 19. The evolutionary conservation of amino acids of TLRs. To calculate the amino acid conservation score, we used the ConSurf web server. For phylogenetic tree reconstruction, we used amino acid multiple sequence alignments of TLR orthologs that are provided by the Ensembl project. A phylogenetic tree was reconstructed using the neighbor-joining algorithm as implemented in the ConSurf server. An evolutionary substitution model was chosen JTT for each TLR as the best-fitted model. The calculation method for the rate of evolution was chosen as Bayesian. Besides, results calculated by the Maximum Likelihood method were similar. The error bar corresponds to the 50% confidence interval of each conservation score estimation from 25% to 75% percentile. For each TLR we indicate the TMD domain according to UNIPROT (yellow background), the hydrophobic JM region according to our analysis (Figure 1) (red background), and the TIR domain according to UNIPROT (blue background). Each bar was colored according to its value of the conservation score from blue (most variable among shown amino acid residues) to red (most conservative among shown amino acid residues). Source data are provided as a Source Data file.

Supplementary References.

1. Triba, M. N., Warschawski, D. E. & Devaux, P. F. Reinvestigation by phosphorus NMR of lipid distribution in bicelles. *Biophys. J.* **88**, 1887–1901 (2005).
2. Mineev, K. S., Nadezhdin, K. D., Goncharuk, S. A. & Arseniev, A. S. Characterization of Small Isotropic Bicelles with Various Compositions. *Langmuir* **32**, 6624–6637 (2016).
3. Wimley, W. C. & White, S. H. Experimentally determined hydrophobicity scale for proteins at membrane interfaces. *Nat. Struct. Biol.* **3**, 842–848 (1996).
4. Mineev, K. S. *et al.* Spatial structure of TLR4 transmembrane domain in bicelles provides the insight into the receptor activation mechanism. *Sci Rep* **7**, 6864 (2017).
