# Supplementary material for: The architecture of transmembrane and cytoplasmic juxtamembrane regions of Toll-like receptors
Source: Nat Commun. 2023 Mar 17;14:1503. doi: 10.1038/s41467-023-37042-6 (PMC10023784; doi:10.1038/s41467-023-37042-6)
Supplement: Supplementary file 3 — Reporting Summary [file 41467_2023_37042_MOESM3_ESM.pdf]

nature portfolio

Corresponding author(s): Konstantin Mineev

Last updated by author(s): Feb 2, 2023

Reporting Summary

Nature Portfolio wishes to improve the reproducibility of the work that we publish. This form provides structure for consistency and transparency in reporting. For further information on Nature Portfolio policies, see our Editorial Policies and the Editorial Policy Checklist.

Statistics

For all statistical analyses, confirm that the following items are present in the figure legend, table legend, main text, or Methods section.

n/a	Confirmed	
☐	☒	The exact sample size (*n*) for each experimental group/condition, given as a discrete number and unit of measurement
☐	☒	A statement on whether measurements were taken from distinct samples or whether the same sample was measured repeatedly
☐	☒	The statistical test(s) used AND whether they are one- or two-sided<br>*Only common tests should be described solely by name; describe more complex techniques in the Methods section.*
☒	☐	A description of all covariates tested
☒	☐	A description of any assumptions or corrections, such as tests of normality and adjustment for multiple comparisons
☐	☒	A full description of the statistical parameters including central tendency (e.g. means) or other basic estimates (e.g. regression coefficient) AND variation (e.g. standard deviation) or associated estimates of uncertainty (e.g. confidence intervals)
☐	☒	For null hypothesis testing, the test statistic (e.g. *F*, *t*, *r*) with confidence intervals, effect sizes, degrees of freedom and *P* value noted<br>*Give P values as exact values whenever suitable.*
☒	☐	For Bayesian analysis, information on the choice of priors and Markov chain Monte Carlo settings
☒	☐	For hierarchical and complex designs, identification of the appropriate level for tests and full reporting of outcomes
☒	☐	Estimates of effect sizes (e.g. Cohen's *d*, Pearson's *r*), indicating how they were calculated

Our web collection on statistics for biologists contains articles on many of the points above.

Software and code

Policy information about availability of computer code

Data collection	Topspin 3.0 (Bruker Biospin) software was used to collect the NMR data. GROMACS 2021.5 was used to run the MD simulations. Analytik Jena qPCRsoft 4.0 was used to collect the qPCR data. Gen5 microplate reader software was used to collect the SEAP data. BD CSample Plus Software (version 1.0)was used to collect the data of flow cytometry.
Data analysis	CARA 1.9.3, CYANA 3.97.8, TALOS-N 4.12, qMDD 3.2, MOLMOL 2K2.0 and PyMOL 2.5.2 were used to analyze the NMR data and protein structures. Analytik Jena qPCRsoft 4.0 and GraphPad Prim 8.0.1 were used to analyze the qPCR data. GraphPad Prim 8.0.1 were used to analyze the SEAP data. FlowJo v.10 was used to analyze the data of flow cytometry.

For manuscripts utilizing custom algorithms or software that are central to the research but not yet described in published literature, software must be made available to editors and reviewers. We strongly encourage code deposition in a community repository (e.g. GitHub). See the Nature Portfolio guidelines for submitting code & software for further information.

Data

Policy information about availability of data

All manuscripts must include a data availability statement. This statement should provide the following information, where applicable:

- Accession codes, unique identifiers, or web links for publicly available datasets
- A description of any restrictions on data availability
- For clinical datasets or third party data, please ensure that the statement adheres to our policy

The coordinates corresponding to the protein structures in this work have been deposited in the Protein Data Bank under accession code 8AR0 for TLR2tmjm, 8AR1 for TLR3tmjm, 8AR2 for TLR5tmjm and 8AR3 for TLR9tmjm. The file with the source data for Figures in the main text and Supplementary materials is available as a Source data archive. All other data are available upon request to the corresponding authors.

Human research participants

Policy information about studies involving human research participants and Sex and Gender in Research.

Reporting on sex and gender	not applicable
Population characteristics	not applicable
Recruitment	not applicable
Ethics oversight	not applicable

Note that full information on the approval of the study protocol must also be provided in the manuscript.

Field-specific reporting

Please select the one below that is the best fit for your research. If you are not sure, read the appropriate sections before making your selection.

☒ Life sciences ☐ Behavioural & social sciences ☐ Ecological, evolutionary & environmental sciences

For a reference copy of the document with all sections, see nature.com/documents/nr-reporting-summary-flat.pdf

Life sciences study design

All studies must disclose on these points even when the disclosure is negative.

Sample size	No statistical methods were used to predetermine sample size. Where required for statistical analyses, sample sizes were determined based on established practice and according to accepted standards in this field: Biological triplicates (at least 3 independent experiments). Sample-size of each experiment was described in figure legends.
Data exclusions	No data points were excluded from the analysis.
Replication	To ensure reproducibility of the findings, for all cell assays, at least three independent biological replicates were performed.
Randomization	Cells were distributed randomly between the experiments
Blinding	Blinding was not relevant to the experiments during data collection and analysis, because experiments were frequently done by the same investigators that might be influenced by bias from the investigator. Matching samples were collected and analyzed under the same conditions.

Reporting for specific materials, systems and methods

We require information from authors about some types of materials, experimental systems and methods used in many studies. Here, indicate whether each material, system or method listed is relevant to your study. If you are not sure if a list item applies to your research, read the appropriate section before selecting a response.

Materials & experimental systems

n/a	Involved in the study
☐	☒ Antibodies
☐	☒ Eukaryotic cell lines
☒	☐ Palaeontology and archaeology
☒	☐ Animals and other organisms
☒	☐ Clinical data
☒	☐ Dual use research of concern

Methods

n/a	Involved in the study
☒	☐ ChIP-seq
☐	☒ Flow cytometry
☒	☐ MRI-based neuroimaging

Antibodies

Antibodies used	FITC anti-TLR1 antibody (Abcam, ab59702) and anti-TLR2 antibody (Abcam, ab59711) for flow cytometry (1:200).
Validation	FITC anti-TLR1 antibody (Abcam, ab59702) was validated by flow cytometric analysis of THP1 cells (Validation information on the manufacturer's website).<br>FITC anti-TLR2 antibody (Abcam, ab59711) was verified in multiple previous publications (PMID: 26860188, 21998404 and 22563430).

Eukaryotic cell lines

Policy information about cell lines and Sex and Gender in Research

Cell line source(s)	HEK Blue 293 cells were purchased from InvivoGen. **(hkb-null2)**
Authentication	HEK Blue 293 cells have been autentified using STR genotyping
Mycoplasma contamination	Cells were tested by a MycoBlue Mycoplasma Detector (Vazyme Biotech, Nanjing, China) to exclude Mycoplasma contamination before experiments.
Commonly misidentified lines<br>(See ICLAC register)	No commonly misidentified lines were used in this study.

Flow Cytometry

Plots

Confirm that:

☒ The axis labels state the marker and fluorochrome used (e.g. CD4-FITC).

☒ The axis scales are clearly visible. Include numbers along axes only for bottom left plot of group (a 'group' is an analysis of identical markers).

☒ All plots are contour plots with outliers or pseudocolor plots.

☒ A numerical value for number of cells or percentage (with statistics) is provided.

Methodology

Sample preparation	HEK Blue 293 cells co-expressed wild-type or chimera of human TLR1 and TLR2 were seeded at a density of 5 × 105 cells/ml in 6-well plates. After 24 h incubation, cells were washed with phosphate-buffered saline (PBS) and subsequently digested with 0.25% trypsin (without EDTA). Cells were collected in a 1.5 ml centrifuge tube and washed three times with 1 ml ice-cold PBS. Cells were then stained with FITC anti-TLR1 antibody (Abcam, ab59702) or FITC anti-TLR2 antibody (Abcam, ab59711) at 1/200 dilution for 30 min on ice in dark. Stained cells were washed twice with ice-cold PBS and analyzed by BD Accuri C6 Plus flow cytometry (BD Life Sciences).
Instrument	BD Accuri C6 Plus flow cytometry.
Software	The data on flow cytometry were collected using BD CSample Plus Software and analyzed using FlowJo v.10.
Cell population abundance	At least 10000 cells were acquried per sample.
Gating strategy	Live single cells were gated based on scatter to exclude cell debris.

☒ Tick this box to confirm that a figure exemplifying the gating strategy is provided in the Supplementary Information.
